# Chain length-dependent mitochondrial toxicity of perfluoroalkyl carboxylic acids: insights from Mito Tox Index evaluation

**DOI:** 10.3389/ftox.2025.1582891

**Published:** 2025-07-15

**Authors:** Yoonseok Kam, Lisa Winer, Natalia Romero

**Affiliations:** Cell and Biomolecular Analysis Division, Agilent Technologies, Lexington, MA, United States

**Keywords:** PFAS (per- and polyfluoroalkyl substances), mitochondrial dysfunction, cell metabolism and bioenergetics, *in vitro* toxicity assay, real time cell analysis

## Abstract

**Introduction:**

Per- and polyfluoroalkyl substances (PFAS) are persistent environmental contaminants that accumulate in living organisms, posing significant human health risks. The toxicity mechanisms of PFAS include mitochondrial dysfunction and bioenergetic failure.

**Methods:**

This study evaluates the structure-activity relationship of PFAS compounds with mitochondrial toxicity by comparing the Mito Tox Index (MTI) of perfluoroalkyl carboxylic acids (PFCAs) varying carbon chain lengths. The MTI quantifies the extent to which substances disrupt mitochondrial function by distinguishing between mitochondrial inhibition and uncoupling. This was followed by an assessment of the effect of PFCAs on total cellular bioenergetics and impedance-based real time cell viability measurement.

**Results and discussion:**

Both inhibition and uncoupling MTI values increased with the chain length of PFCAs and severe mitochondrial inhibition was observed when uncoupling was maximized by PFCAs containing seven or more carbons within hours of exposure. The mitochondrial toxicity corresponded well to the bioenergetic failure measured by real-time ATP production rates. In contrast, there was a substantial difference between cytotoxicity and mitochondrial toxicity, despite a common trend of increased toxicity with longer chain lengths. The results suggest that PFCA-induced mitochondrial dysfunction is a key mechanism of PFAS-mediated cellular damage, primarily driven by proton leak-mediated ETC uncoupling, leading to impaired mitochondrial energy production. It also implies that MTI-based mitochondrial toxicity evaluation increases data precision in comparing PFAS effects on mitochondrial function, even identifying the mode of action, which is expected to improve in vitro toxicity prediction models.

## Introduction

Mitochondria produce ATP via the electron transport chain (ETC), a process essential for energy metabolism. This unique cellular organelle with complex membrane structure and morphology is increasingly recognized not just as the powerhouse of the cell, but more importantly as a regulatory center for eukaryotic cells. It plays crucial roles for maintaining cellular homeostasis and survival and many pivotal roles in cellular regulation including signaling, cellular differentiation, and apoptosis (Picard and Shirihai, 2022). This vital organelle is also a target for drug toxicity and environmental toxicity, making it a focal point in toxicological studies (Ehrlich et al., 2023; Nadanaciva and Will, 2011; Reddam et al., 2022).

PFAS, often referred to as “forever chemicals,” are persistent environmental pollutants that accumulate in living organisms and pose significant health risks to humans (Deepika et al., 2022; Schiavone and Portesi, 2023). These risks include cancer, reproductive and developmental defects, immune system alterations, hormone disruption, liver damage, and more (Zheng et al., 2024). Thousands of PFAS have been identified, and extensive research is ongoing to understand their mechanisms of action and predict health risks. While intensive studies on some legacy PFAS, such as Perfluorooctanoic acid (PFOA) and perfluorooctane sulfonate (PFOS), have provided better insights into their mechanisms, many aspects remain unknown and require further investigation due to the vast number of PFAS variants.

One of the early studies on the structure–cytotoxicity *in vitro* relationship suggested a strong correlation between the chain length and the induced cytotoxicity (Kleszczynski et al., 2007). The study was followed by reports showing a good correlation of PFAS-induced mitochondrial dysfunction and ROS production. As a common outcome of mitochondrial dysfunction, the chain-length dependent ROS increase can be a result from mitochondrial inhibition caused PFAS (Amstutz et al., 2022; Kleszczynski and Skladanowski, 2009; Kleszczynski et al., 2009).

The Mito Tox Index (MTI) is a standardized numerical scale used to quantify the extent of chemical-induced mitochondrial toxicity. It is derived from measurements of mitochondrial oxygen consumption rate (OCR) obtained using the Agilent Seahorse XF platform. This study evaluated the contribution of PFAS-chain length on mitochondrial toxicity by comparing the MTIs of PFASs with different chain length. A group of PFCAs containing 3 to 10 carbons were selected as the model compound set to limit the variables to physical length and exclude other molecular contributions. In addition to the mitochondrial toxicity, the effect of the compounds in total cellular bioenergetics and cellular cytotoxicity was also investigated. HepG2, a widely used human liver cancer cell line in both hepatotoxicity and mitochondrial toxicity tests was used as in the previous reports (Amstutz et al., 2022; Nadanaciva et al., 2012).

## Materials and methods

### Cells and materials

HepG2 cell line was obtained from ATCC and cultured in low-glucose DMEM (Gibco) supplemented with 2 mM GlutaMAX (Gibco) and 10% serum (HyClone). PFCAs containing different carbon lengths were purchased from Sigma-Aldrich as summarized in Table 1. The assay kits and reagents required for Mito Tox assay, real-time ATP production rate measurement, and real-time cell analysis (RTCA) were purchased from Agilent Technologies, Inc. and used according to the manufacturer’s manual as described briefly below.

**TABLE 1:** PFCAs used for evaluation.

PFCAs	Abbreviation	Chain length	Product no.
Perfluoropropionic acid	PFPA	C3	245917
Perfluorobutyric acid	PFBA	C4	164194
Perfluorohexanoic acid	PFHxA	C6	43809
Perfluoroheptanoic acid	PFHA	C7	43996
Perfluorooctanoic acid	PFOA	C8	171468
Perfluorononanoic acid	PFNA	C9	91977
Perfluorodecanoic acid	PFDA	C10	43929

Liquid PFPA and PFBA were diluted in either XF assay medium or HepG2 culture medium to prepare 2 mM stock solutions. These stock solutions were pH-adjusted to 7.4, filtered, and stored at 4°C for up to 1 week. Stock solutions for other PFCAs (PFHA, PFHxA, PFOA, PFNA, PFDA) were prepared at 500 mM in methanol and stored at −20°C. Prior to treatment, PFCA stock solutions were further diluted in XF assay medium or cell culture medium.

### Mitochondrial toxicity

The MTI for inhibition and uncoupling of PFCAs was assessed using the Seahorse XF Mito Tox assay kit by Seahorse XF Pro analyzer (Agilent). Briefly, HepG2 cells were seeded in Agilent Seahorse XF Pro M cell culture microplates (Agilent) at a density of 2× 10^4^ cells per well and cultured for 24 h. The following day, the cells were washed twice with XF assay medium (Agilent Seahorse XF DMEM medium, pH 7.4, supplemented with 10 mM Agilent Seahorse XF glucose solution, 1 mM Agilent Seahorse XF pyruvate solution, and 2 mM Agilent Seahorse XF glutamine solution) and incubated at 37°C without CO_2_ for 60 min in the presence of vehicle, PFCAs, or Rot/AA prior to measuring OCR with the Agilent XF Pro analyzer. All MTIs were automatically calculated using Seahorse Analytics, a cloud-based XF software.

**FIGURE 1:**
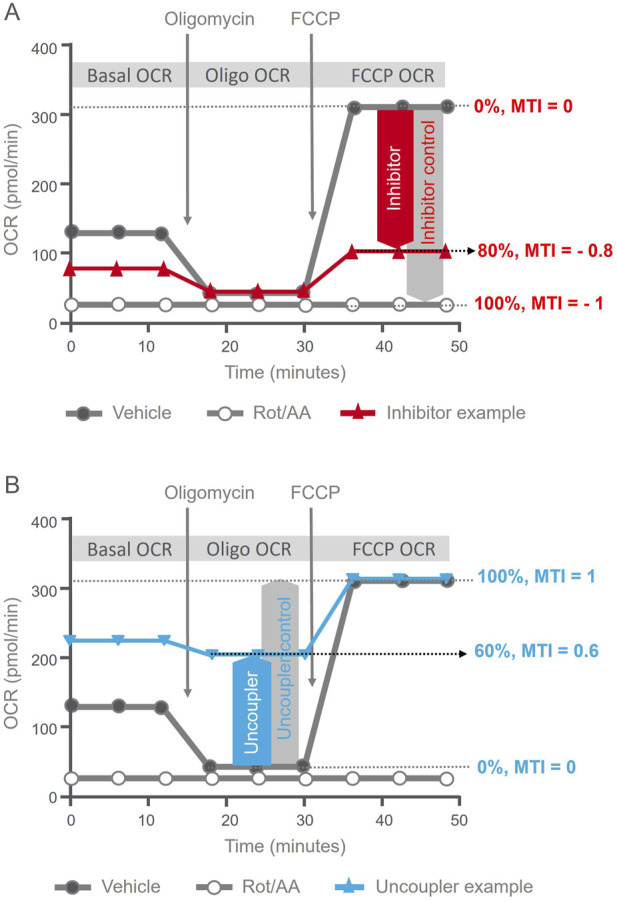
Illustrative summary of MTI calculation principles. Agilent Seahorse XF Mito Tox assay kinetic OCR profiles illustrating the definition of MTI and criteria used to assess the mode and the magnitude of mitochondrial toxicity. **(A)** MTI definition for inhibition; the red line illustrates example kinetic OCR trace of a compound showing mitochondrial toxicity via inhibition, with an MTI = - 0.8. **(B)** MTI definition for uncoupling; the blue line illustrates example kinetic OCR trace of a compound showing mitochondrial toxicity via uncoupling, with an MTI = 0.6.

Figure 1 schematically illustrates the principle of MTI calculation. The MTI for mitochondrial inhibitors is calculated by comparing the maximal OCR achieved by the injection of the validated uncoupler Carbonyl cyanide-p-trifluoromethoxy phenylhydrazone (FCCP) of the XF Mito Tox assay kit in the cells exposed to the test compound versus of vehicle control, and scaled from 0 to –1 (Figure 1A). From the same OCR kinetic data, the MTI for uncouplers calculated by comparing the proton leaked OCR portion detected after the injection of oligomycin, a validated ATP synthase inhibitor, in cells exposed to the test compound versus the FCCP-induced uncoupling effect of the vehicle group (Figure 1B) and scaled from 0 to 1. The detailed principle of MTI calculation and the corresponding formulas are available on the manufacturer’s resource page (Kam et al., 2022; Rogers et al., 2023).

### Bioenergetic evaluation

The changes in the bioenergetic status of HepG2 cells were assessed using the XF Real-time ATP Rate assay kit (Agilent). The cell seeding and sample preparation were identical to those used in the XF Mito Tox assay. To assess ATP production rates, changes in real-time mitochondrial OCR and glycolytic proton efflux rate (PER) were measured using the Seahorse XF Pro, following the manufacturer’s user manual with sequential administration of oligomycin and a rotenone/antimycin A mix was performed. The OCR and PER kinetic data were then converted to mitochondrial and glycolytic ATP production rates, respectively, as previously described (Romero et al., 2018). Briefly, ATP-coupled OCR was obtained from the change in OCR induced by oligomycin, an ATP synthase inhibitor, and converted to mitochondrial ATP production rate using the P/O ratio (the stoichiometry of ATP phosphorylated per atom of oxygen reduced). An experimentally validated average P/O value of 2.75 was applied. The glycolytic ATP production rate was directly obtained from the basal PER, subtracting the mitochondrial contribution to PER via CO_2_ production. The total ATP production rate is the sum of the ATP production rates from glycolysis and mitochondrial ATP production. All rates were automatically calculated and analyzed using Seahorse Analytics software.

### Real-time cell index-based cytotoxicity evaluation

Real-time changes in the Cell Index using the xCelligence RTCA MP system (Agilent) were monitored to evaluate the impact of PFCAs on cell viability, following the manufacturer’s guidelines. Briefly, E-Plate View 96 (Agilent) was preloaded with 50 µL/well of prewarmed growth media, and a background measurement was performed on the RTCA MP system. HepG2 cells were resuspended in growth media at a density of 2 x 10^5^ cells/mL, and 100 µL of the cell suspension was dispensed into each well of the E-Plate resulting in 2 x 10^4^ cells/well and the final media volume was adjusted to 150 µL/well. The cell-containing E-Plate was returned to the RTCA MP system, and impedance was measured every 30 min for approximately 24 h prior to PFCs administration. The PFCA working solution was prepared at 4x concentration in the growth medium and added to the cells at 50 µL/well resulting in a final volume of 200 µL/well. Impedance measurements were resumed, and data were collected every hour for at least 72 h.

### Experimental design and data analysis

All data were obtained from three independent experiments. Each data point represents at least three replicates and is presented as mean ± S.D. Statistical significance was determined by two-way ANOVA between the experimental groups with multiple comparisons.

## Results

### MTI-based evaluation of chain-length correlation to mitochondrial toxicity

**FIGURE 2:**
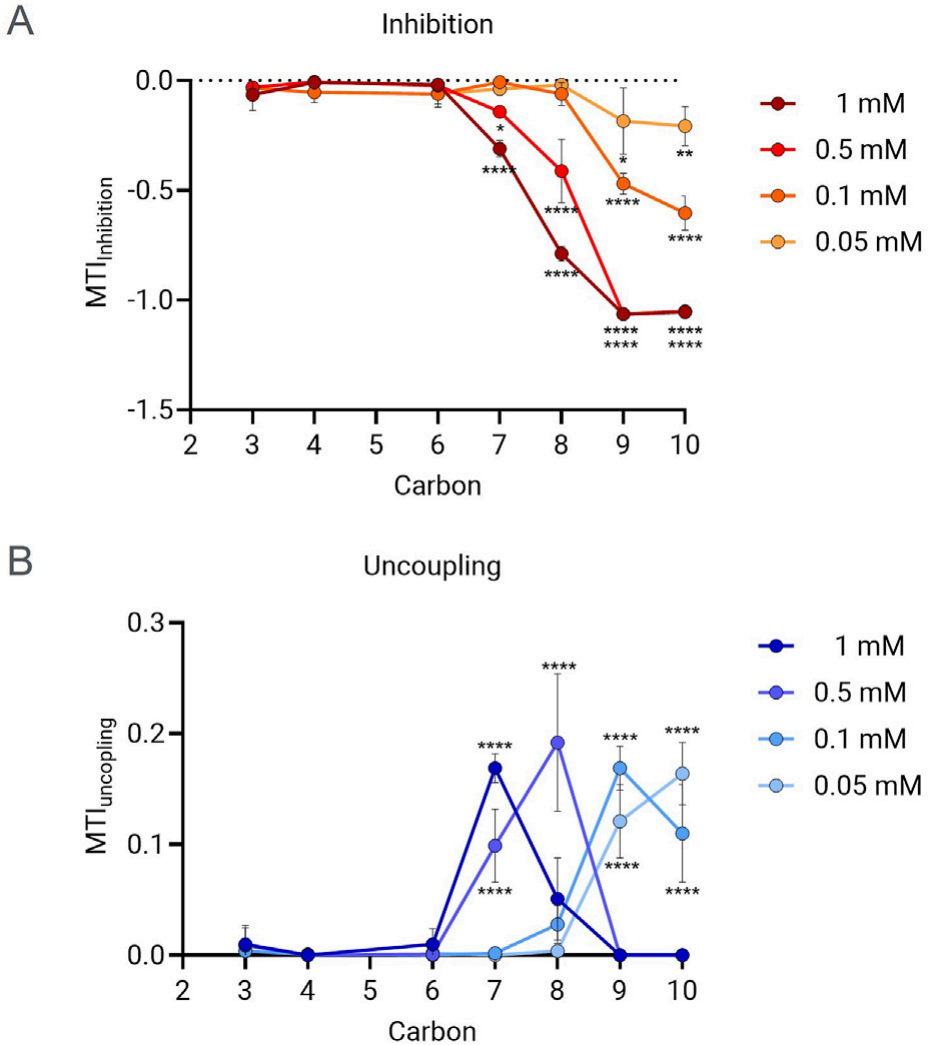
MTI comparison between PFCAs of different chain lengths. The MTI for ETC inhibition **(A)** and uncoupling **(B)** were measured in the presence of PFCAs of different carbon chain length at different concentrations: 0.05, 0.1, 0.5 and 1 mM. (n = 3, Mean ± S.D.). *P ≤ 0.05, **P ≤ 0.005, ****P ≤ 0.00005 by two-way ANOVA with multiple comparisons to the PFHxA (C6) within the same concentration.

Mitochondrial toxicity study in HepG2 cells showed that PFCAs compounds induce both mitochondrial inhibition and uncoupling, with strong chain length and concentration dependency. As shown in Figure 2A, longer PFCAs exhibited a greater magnitude of inhibition MTI at the same concentration. A clear dose-dependent increase in the inhibitory effect was evident for PFCAs with 7 or more carbons. No significant acute inhibitory mitochondrial toxicity was observed when the carbon chain length was less than 7, even at a concentration of 1 mM.

The relationship between chain length and uncoupling effect was also identified. As shown in Figure 2B, a lower PFCAs concentration was required to achieve the maximal uncoupling effect for longer chain lengths. For example, 0.05–0.1 mM was sufficient to show an MTI higher than 0.1 for PFNA and PFDA, while 0.5 mM was required for PFOA. No significant uncoupling was induced by PFCAs with fewer than 7 carbons, similar to what was observed for the inhibitory effect.

**FIGURE 3:**
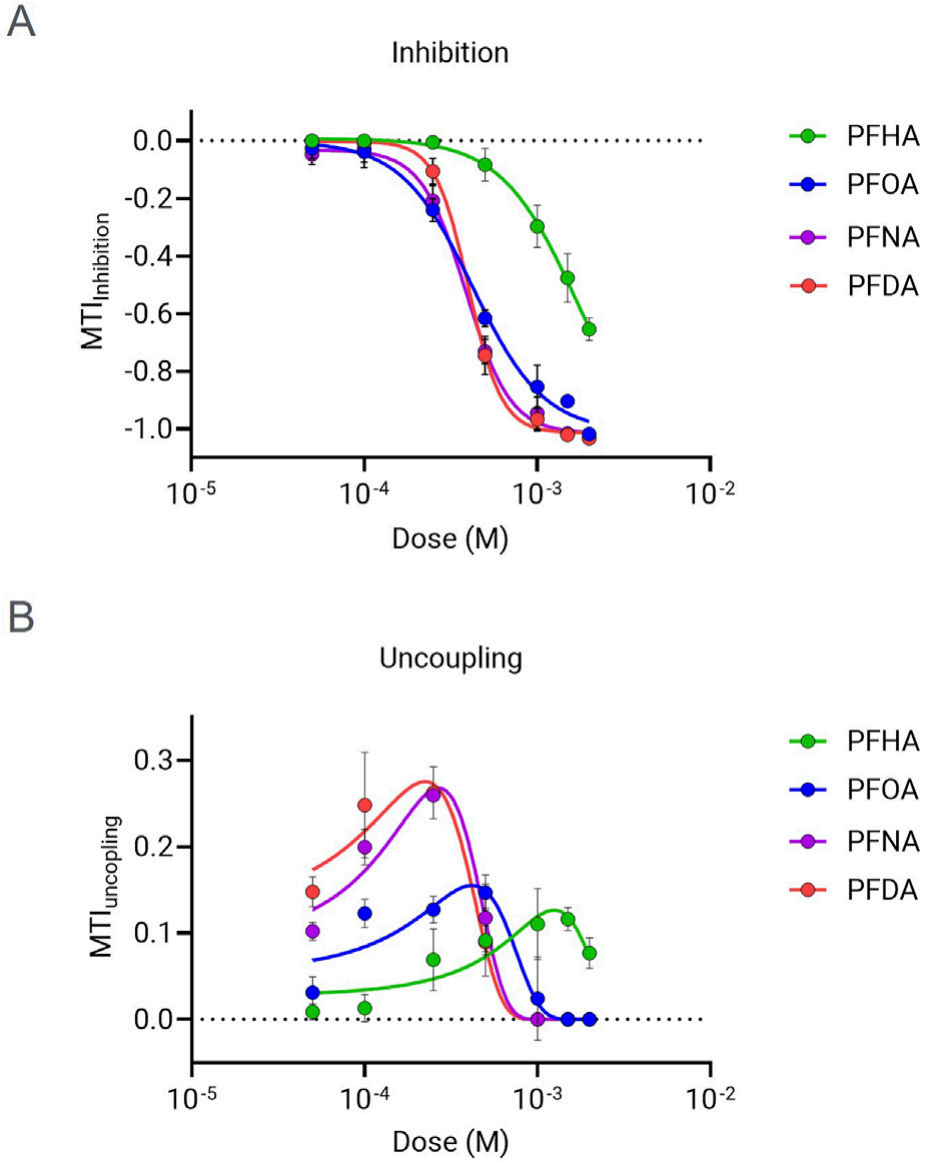
Dose response analysis of MTI for selected PFCAs. The MTI for ETC inhibition **(A)** and uncoupling **(B)** were measured in the presence of PFCAs at various concentrations from 50 μM to 2 mM. (n = 4, Mean ± S.D.).

The mitochondrial toxicity of PFCAs with 7 or more carbons, which exhibited significant mitochondrial toxicity in Figure 2, was further evaluated by determining the dose of 50% inhibition (IC_50_) from the MTI dose-response data. As shown in Figure 3A, PFOA, PFNA, and PFDA exhibited a strong dose-dependent ETC inhibitory effect compared to PFHA. Although PFOA showed a slightly milder effect compared to PFNA and PFDA, there was no significant difference in the IC_50_ values (PFDA (C10) = PFNA (C9) > PFOA (C8) >> PFHA (C7)).

Many mitochondrial uncoupler compounds have a dose dependent bell shape effect, with OCR increasing with rising uncoupler concentration until it reached the “optimal’ uncoupling dose, but then higher concentration inducing decreases in OCR due to additional mitochondrial inhibitory effect (Kenwood et al., 2014). The four mitochondria-inhibiting PFCAs showed the typical dose-response bell shape pattern (Figure 3B). For that reason, Gaussian curve fitting was applied to identify the dose that results in maximal respiration rate. As summarized in Table 2, the dose showing maximal uncoupling effect is lower than the corresponding IC_50_ values of MTI for inhibition. This implies that the uncoupler effect of PFCA is the primary mode of the mitochondrial toxicity, which results in respiratory chain failure when the dose exceeds the “optimal” concentration, or the uncoupling molecule is accumulated in the cells by chronic exposure.

**TABLE 2:** IC_50_ values obtained for mitochondrial toxicity, cytotoxicity and compound concentration that induces maximal uncoupling for the PFCAs compounds studied that exhibited toxicity.

PFAS	Chain length	MTI _inhibition_ IC_50_ (mM)	MTI _uncoupling_ peak (mM)	Cell index _AUC 48 h_ IC_50_ (mM)
PFHA	C7	1.60	1.25	0.60
PFOA	C8	0.42	0.42	0.24
PFNA	C9	0.39	0.27	0.24
PFDA	C10	0.40	0.22	0.52

### Evaluation of chain-length correlation to bioenergetic failure

**FIGURE 4:**
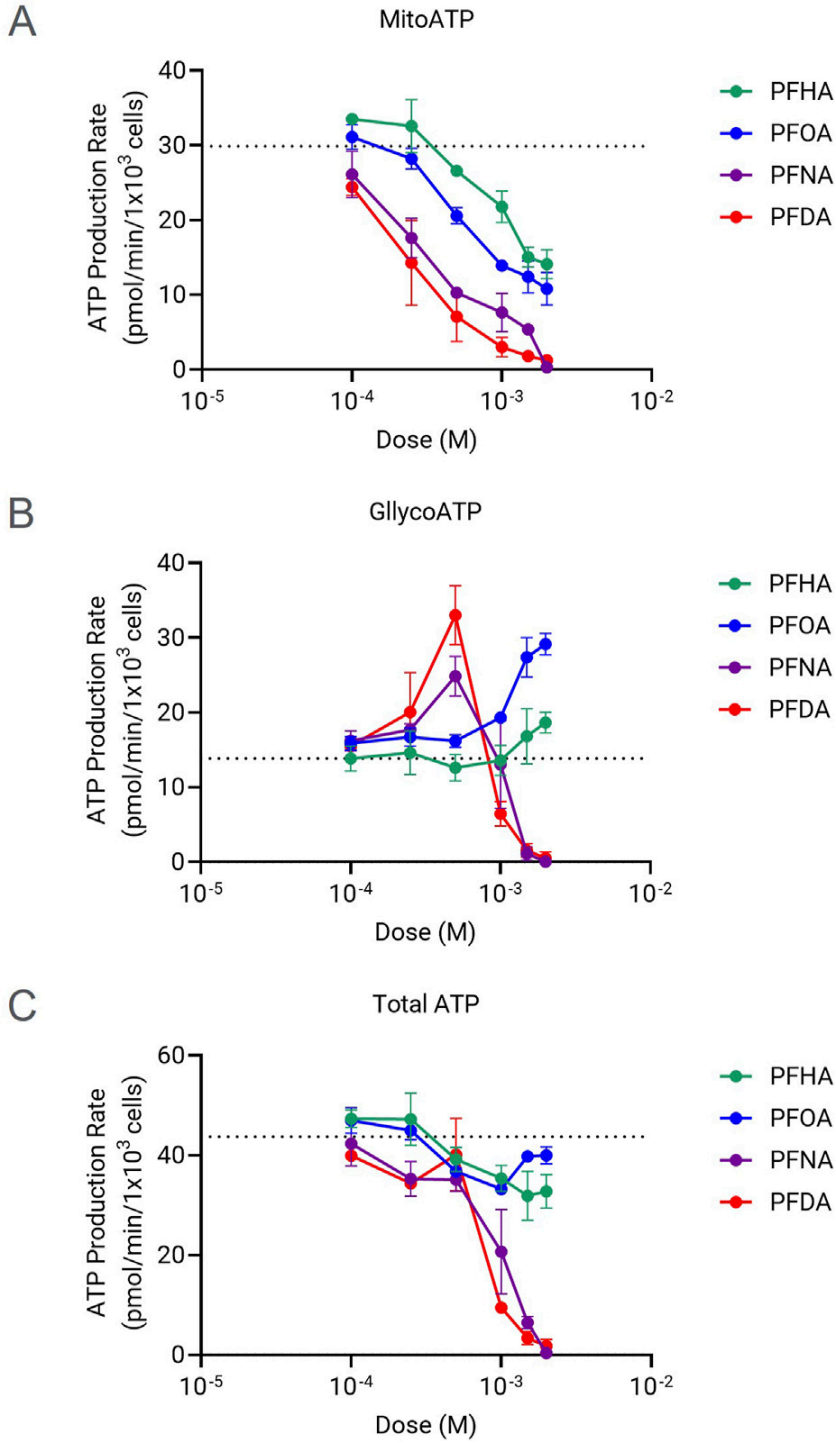
Changes in the ATP production rates induced by PFCAs with 7 or more carbons. Mitochondrial ATP production rates **(A)** glycolytic ATP production rate **(B)**, and total ATP production rates**(C)** of HepG2 cells exposed for 1 h to different concentrations of the PFCAs compounds (n = 4, Mean ± S.D.).

Cellular energy depletion also could be a critical functional defect caused by mitochondrial failure, although ROS production is the most well-proposed mechanism of PFAS toxicity mediated by mitochondrial dysfunction (Iba et al., 2025; Mihajlovic and Vinken, 2022). The chain-length relation to the ROS production was reported previously (Amstutz et al., 2022). In this study, the real-time ATP production rate changes caused by PFCAs were examined to find if there is any chain-length related bioenergetic failure along with mitochondrial dysfunction. The mitochondrial ATP production rate decreased in a dose-dependent manner for all four mitochondrial-toxic PFCAs as expected (Figure 4A) and the impact of PFCAs on total cellular ATP production rate also correlated well with chain length, ranked as PFDA (C10) = PFNA (C9) >> PFOA (C8) > PFHA (C7). However, there are some distinctions between the effect of PFCAs on total ATP production rate and the effect on mitochondrial toxicity. Particularly, as shown in Figure 4C, the total ATP production rate was maintained well even at high PFCA doses, concentrations that caused a significant impact on the mitochondrial function in Figure 3. This maintenance in bioenergetic state is mainly due to a compensatory increase in glycolysis activity to sustain ATP production. For example, while PFOA and PFHA significantly downregulated mitochondrial ATP production rates, the elevated glycolytic ATP production compensated it with small effect in total cellular bioenergetics at least during short exposure (Figure 4B). The same compensation occurred in PFNA- or PFDA-treated cells up to 0.5 mM. However, the compensatory effect was no longer observed at higher concentrations, as indicated by the drop in the ATP production rates at concentrations higher than 0.5 mM (Figure 4C) indicating that at higher concentrations, cellular bioenergetic status is compromised.

### Cell index-based evaluation of chain-length correlation to cytotoxicity

**FIGURE 5:**
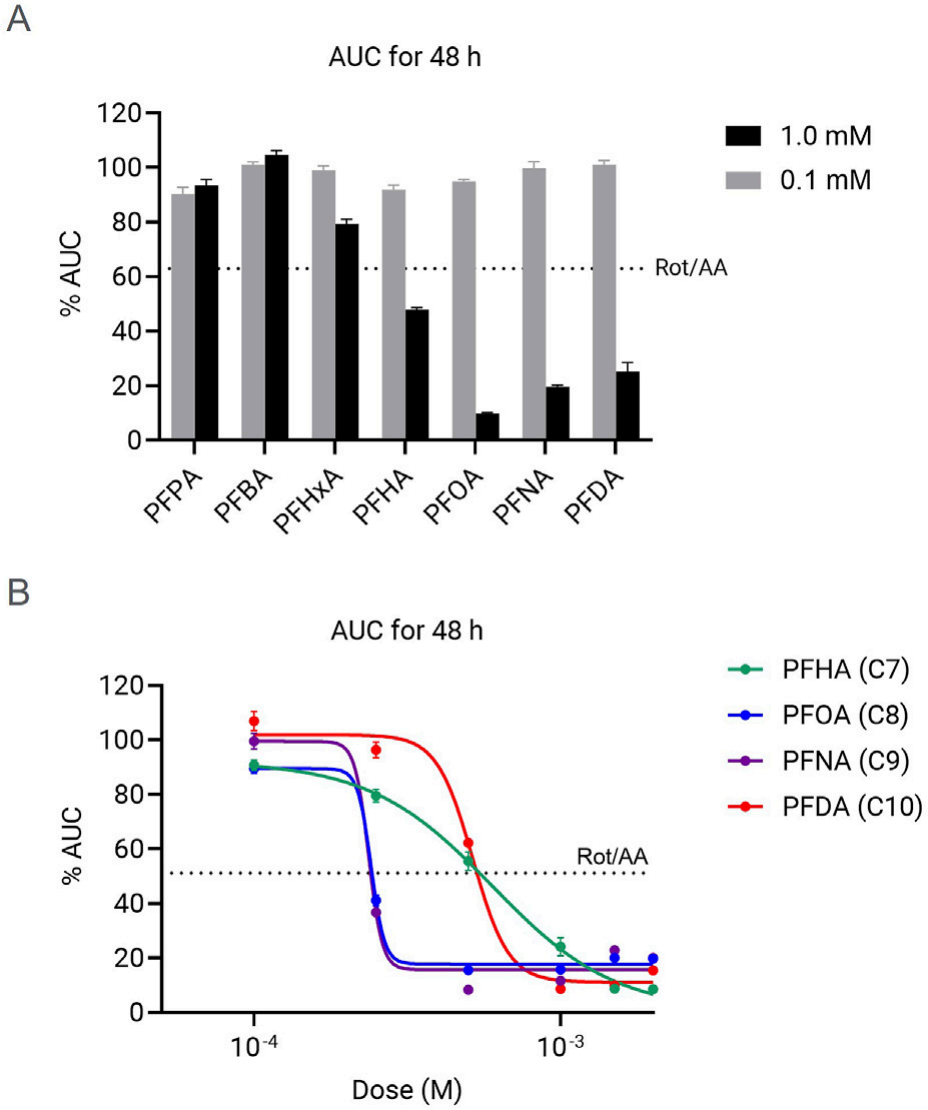
Chain-length and dose-dependent cytotoxicity caused by various PFCAs. **(A)** AUC of normalized cell index up to 48 h after the PFCA treatment. **(B)** PFCA does response of normalized cell index change by AUC to 48 h (n = 4, Mean ± S.D.).

We then investigated the effect of long exposure of HepG2 cells to PFCAs using real-time impedance measurement to determine cytotoxicity (Supplementary Figure S1). For better comparison of the magnitude of the induced toxicity, the AUC (area under the curve) of the Cell Index kinetics up to 48 h was calculated. The AUC at 48 h is representative of the cytotoxicity observed considering other time exposure intervals between 6–72 h (Supplementary Figure S2; Supplementary Table S1). As a reference, cytotoxicity induced by ETC complex I and III inhibition by 0.5 µM rotenone and antimycin A mix (Rot/AA) was used as a reference for mitochondrial dysfunction-induced cytotoxicity. Similar to what we observed for short exposure PFCAs-dependent mitochondrial toxicity, there was no significant cytotoxicity observed even in the presence of high dose (1 mM) for PFCAs compounds shorter than 6 carbon length. No cytotoxicity was detected either by low dose (0.1 mM) PFCAs, regardless of the chain length, up to 72 h (Figure 5A). All mitochondrial toxic PFCAs with 7 or more carbons showed significant cytotoxicity at 1 mM. In addition, PFHxA, with 6 carbons length, showed a moderate decrease in the Cell Index, although it did not show mitochondrial toxicity.

Similar to what we observed for mitochondrial toxicity, there is a clear relationship between chain-length and cytotoxicity, but the dose-response data indicates a substantial difference in the ranking of the compounds (Figure 5A). While for mitochondrial toxicity and bioenergetic failure, chain length directly correlates with induced dysfunction, in the case of impedance-based cytotoxicity measurements the compounds with 8- and 9 carbons length showed the highest cytotoxic effect and slightly decrease for the 10-C length compound (PFNA (C9) = PFOA (C8) > PFDA (C10) > PFHA (C7), Figure 5B). Additionally, the impact of 4 mitochondrially toxic PFCAs at the doses higher than IC_50_ doses was higher than Rot/AA control group in which the mitochondrial respiration is completed suppressed. This implies that cell death caused by long-chain PFCAs at high concentration is mediated by multiple modes of action beyond mitochondrial dysfunction.

## Discussion

Advances in technology for screening the effects of large libraries of compounds on cellular function necessitate the adoption of simple outputs that facilitate easy comparisons of compound potency. Over the past 20 years, the Mitochondrial Stress Test has been widely used to evaluate the effects of compounds on mitochondrial function by measuring the extracellular flux of oxygen consumption rates. However, studies involving large compound libraries require extensive data analysis and interpretation. The use of unitless and indexed parameters for toxicity quantification, such as the MTI, allows precise comparisons of the impact of chemical compounds on cellular mitochondrial dysfunction and provides a straightforward method for toxicity ranking. Additionally, incorporating internal control groups enhances comparability between studies, reducing dependency on experimental settings. Conversely, Cell Index-based real-time cytotoxicity monitoring offers a comprehensive view of cell death induced by toxins or drugs by simultaneously considering both time and dose factors.

Various studies used *in vitro* culture models to predict and to understand the mechanism of action of PFAS-toxicity. They proposed ROS production which results in oxidative DNA damage as one of the major pathways (Chen et al., 2018; Eriksen et al., 2010; Han et al., 2018; Panaretakis et al., 2001; Xu et al., 2019). However, it is not well elucidated how PFAS induce ROS generation nor at what extent ROS production-associated toxicological mechanism exerts the cytotoxic outcome. Better understanding of toxicological effects and more accurate identification of cellular targets is important to assessing toxicity of emerging PFAS. In addition, more quantitative and comparable analysis is needed to build a more accurate and reliable toxicity model applicable for a broad range of PFAS compounds.

Mitochondrial dysfunction is a well-known source of ROS production (Chen et al., 2018). The chain-length correlation of mitochondrial toxicity of PFAS was more precisely proven in this study by determining MTI, a quantitative parameter of toxicity in agreement with previous report correlating ROS production with PFAS chain-length (Amstutz et al., 2022). Furthermore, it was identified that the core mechanism of action of mitochondrial toxicity could be membrane instability, which tends to be more severe with longer chain molecules. It implies that evaluation of uncoupler activity of PFAS might be a critical factor in assessing PFAS mitochondrial toxicity.

Since the MTI of a drug or toxin is defined as a unitless scale ranging from 0 to 1 or -1, it effectively indexes the measurement of mitochondrial toxicity. This indexing facilitates more straightforward comparisons between different experiments and studies. Additionally, the use of an indexed impact scale offers significant advantages in the development of theoretical prediction models, as it provides a standardized and quantifiable method for assessing and predicting the mitochondrial toxicity of various substances. A similar benefit can be obtained from the Cell Index-based cytotoxicity evaluation. As summarized in Table 2, a more precise comparison between mitochondrial dysfunction and cytotoxicity as well as the chain-length correlation is feasible comparing both indexes.

Even though ROS production is the most prominent pathway leading to cell death upon PFAS-mitochondrial damage, the involvement of other mechanisms responsible for the cytotoxicity cannot be excluded. For example, the bioenergetic failure can be an additional defect contributing to cell death. Glycolysis commonly compensates for the energy deficiency incurred by moderate mitochondrial failure in eukaryotic cells. However, when cells experience sustained and severe mitochondrial dysfunction, total ATP production cannot fully rely on glycolytic activity, resulting in bioenergetic failure as exemplified in Figure 4B.

Although this study demonstrated that the combined application of MTI and cell index provides a better understanding of PFAS toxicity, particularly hepatotoxicity, further investigation is required. For example, while the uncoupler effect is a major driver of mitochondrial toxicity, the possibility of direct hits on ETC components cannot be excluded. The apoptotic function of mitochondria also needs to be considered. An early study correlating chain length to cell death proposed that the release of cytochrome c, along with the mitochondrial permeability transition, is a mechanism of PFAS-induced cell death in addition to bioenergetic failure (Kleszczynski et al., 2009). Intracellular delivery and accumulation are other factors that can change the impact of PFAS toxicity (Nguyen et al., 2024).

Despite the strong chain-length correlation to toxicity, PFDA with 10 carbons consistently showed lower cytotoxicity compared to PFOA or PFNA (Figure 5), as previously reported (Amstutz et al., 2022; Kleszczynski et al., 2009), in contrast to highest mitochondrial toxicity (Figures 2, 3). The delayed cytotoxicity may hint at why PFDA showed relatively lower toxicity among long-chain PFCAs (Supplementary Figure S1). More comprehensive analyses, such as direct ETC flow evaluation using permeabilized cells or isolated mitochondria, may be necessary, especially to understand the effects of chronic exposure to moderate doses that do not cause immediate cell death.

## Data Availability

The raw data supporting the conclusions of this article will be made available by the authors, without undue reservation.

## Supplementary material

SUPPLEMENTARY TABLE S1 IC_50_ of PFCAs for different durations of exposure.

SUPPLEMENTARY FIGURE S1 The cytotoxic effect of different PFCAs was compared by monitoring real-time cell index changes for up to 72 h in the presence of low concentrations (50 and 100 μM, A) or high concentrations (0.5 and 1 mM, B) of PFCAs. (n = 4, Mean ± S.D.).

SUPPLEMENTARY FIGURE S2
**(A)** PFCAs were administered approximately 24 h after seeding as indicated. Comparison of normalized AUC of Cell Index obtained at different durations of compound exposure. **(B)** Dose response curves of cytotoxicity to PFCAs at different durations of compound exposure. (n = 4, Mean ± S.D.).

## References

[B1] Amstutz V. H. Cengo A. Gehres F. Sijm D. Vrolijk M. F. (2022). Investigating the cytotoxicity of per- and polyfluoroalkyl substances in HepG2 cells: a structure-activity relationship approach. Toxicology 480, 153312. 10.1016/j.tox.2022.153312 36075290

[B2] Chen Y. Zhou Z. Min W. (2018). Mitochondria, oxidative stress and innate immunity. Front. Physiol. 9, 1487. 10.3389/fphys.2018.01487 30405440 PMC6200916

[B3] Deepika D. Rovira J. Sabuz O. Balaguer J. Schuhmacher M. Domingo J. L. (2022). Framework for risk assessment of PFAS utilizing experimental studies and in-silico models. Environ. Res. 208, 112722. 10.1016/j.envres.2022.112722 35026182

[B4] Ehrlich V. Bil W. Vandebriel R. Granum B. Luijten M. Lindeman B. (2023). Consideration of pathways for immunotoxicity of per- and polyfluoroalkyl substances (PFAS). Environ. Health 22 (1), 19. 10.1186/s12940-022-00958-5 36814257 PMC9944481

[B5] Eriksen K. T. Raaschou-Nielsen O. Sorensen M. Roursgaard M. Loft S. Moller P. (2010). Genotoxic potential of the perfluorinated chemicals PFOA, PFOS, PFBS, PFNA and PFHxA in human HepG2 cells. Mutat. Res. 700 (1-2), 39–43. 10.1016/j.mrgentox.2010.04.024 20451658

[B6] Han R. Hu M. Zhong Q. Wan C. Liu L. Li F. (2018). Perfluorooctane sulphonate induces oxidative hepatic damage via mitochondria-dependent and NF-κB/TNF-α-mediated pathway. Chemosphere 191, 1056–1064. 10.1016/j.chemosphere.2017.08.070 28939271

[B7] Iba T. Helms J. Maier C. L. Ferrer R. Levy J. H. (2025). Mitochondrial dysfunction is a major cause of thromboinflammation and inflammatory cell death in critical illnesses. Inflamm. Res. 74 (1), 17. 10.1007/s00011-025-01994-w 39806233

[B8] Kam Y. Rogers G. W. Winer L. Schwalfenberg M. Romero N. (2022). A customized XF workflow for detection and characterization of mitochondrial toxicity. Agil. Technol. Appl. Note, 5994–4778EN. Available online at: https://www.agilent.com/cs/library/applications/an-customized-xf-workflow-for-detection-of-mitocondrial-toxicity-5994-4778en-agilent.pdf.

[B9] Kenwood B. M. Weaver J. L. Bajwa A. Poon I. K. Byrne F. L. Murrow B. A. (2014). Identification of a novel mitochondrial uncoupler that does not depolarize the plasma membrane. Mol. Metab. 3 (2), 114–123. 10.1016/j.molmet.2013.11.005 24634817 PMC3953706

[B10] Kleszczynski K. Gardzielewski P. Mulkiewicz E. Stepnowski P. Skladanowski A. C. (2007). Analysis of structure-cytotoxicity *in vitro* relationship (SAR) for perfluorinated carboxylic acids. Toxicol Vitro 21 (6), 1206–1211. 10.1016/j.tiv.2007.04.020 17572060

[B11] Kleszczynski K. Skladanowski A. C. (2009). Mechanism of cytotoxic action of perfluorinated acids. I. alteration in plasma membrane potential and intracellular pH level. Toxicol. Appl. Pharmacol. 234 (3), 300–305. 10.1016/j.taap.2008.10.008 19026671

[B12] Kleszczynski K. Stepnowski P. Skladanowski A. C. (2009). Mechanism of cytotoxic action of perfluorinated acids II. Disruption of mitochondrial bioenergetics. Toxicol. Appl. Pharmacol. 235 (2), 182–190. 10.1016/j.taap.2008.11.021 19135466

[B13] Mihajlovic M. Vinken M. (2022). Mitochondria as the target of hepatotoxicity and drug-induced liver injury: molecular mechanisms and detection methods. Int. J. Mol. Sci. 23 (6), 3315. 10.3390/ijms23063315 35328737 PMC8951158

[B14] Nadanaciva S. Rana P. Beeson G. C. Chen D. Ferrick D. A. Beeson C. C. (2012). Assessment of drug-induced mitochondrial dysfunction via altered cellular respiration and acidification measured in a 96-well platform. J. Bioenerg. Biomembr. 44 (4), 421–437. 10.1007/s10863-012-9446-z 22689143

[B15] Nadanaciva S. Will Y. (2011). Investigating mitochondrial dysfunction to increase drug safety in the pharmaceutical industry. Curr. Drug Targets 12 (6), 774–782. 10.2174/138945011795528985 21275886

[B16] Nguyen T. V. Trang P. N. Kumar A. (2024). Understanding PFAS toxicity through cell culture metabolomics: current applications and future perspectives. Environ. Int. 186, 108620. 10.1016/j.envint.2024.108620 38579451

[B17] Panaretakis T. Shabalina I. G. Grander D. Shoshan M. C. DePierre J. W. (2001). Reactive oxygen species and mitochondria mediate the induction of apoptosis in human hepatoma HepG2 cells by the rodent peroxisome proliferator and hepatocarcinogen, perfluorooctanoic acid. Toxicol. Appl. Pharmacol. 173 (1), 56–64. 10.1006/taap.2001.9159 11350215

[B18] Picard M. Shirihai O. S. (2022). Mitochondrial signal transduction. Cell Metab. 34 (11), 1620–1653. 10.1016/j.cmet.2022.10.008 36323233 PMC9692202

[B19] Reddam A. McLarnan S. Kupsco A. (2022). Environmental chemical exposures and mitochondrial dysfunction: a review of recent literature. Curr. Environ. Health Rep. 9 (4), 631–649. 10.1007/s40572-022-00371-7 35902457 PMC9729331

[B20] Rogers G. W. Winer L. Schwalfenberg M. Romero N. Kam Y. (2023). Principle of mitochondrial toxicity assessment using agilent Seahorse XF solution. Agil. Technol. White Pap., 5994–4732EN. Available online at: https://www.agilent.com/cs/library/whitepaper/public/wp-principle-of-mitochondrial-toxicity-assessment-5994-4732en-agilent.pdf.

[B21] Romero N. Rogers G. Neilson A. Dranka B. P. (2018). Quantifying cellular ATP production rate using agilent Seahorse XF technology. Agil. Technol. White Pap., 5991–9303EN. Available online at: https://www.agilent.com/cs/library/whitepaper/public/whitepaper-quantify-atp-production-rate-cell-analysis-5991-9303en-agilent.pdf.

[B22] Schiavone C. Portesi C. (2023). PFAS: a review of the state of the art, from legislation to analytical approaches and toxicological aspects for assessing contamination in food and environment and related risks. Appl. Sci. 13 (11), 6696. 10.3390/app13116696

[B23] Xu M. Wan J. Niu Q. Liu R. (2019). PFOA and PFOS interact with superoxide dismutase and induce cytotoxicity in mouse primary hepatocytes: a combined cellular and molecular methods. Environ. Res. 175, 63–70. 10.1016/j.envres.2019.05.008 31103794

[B24] Zheng J. Liu S. Yang J. Zheng S. Sun B. (2024). Per- and polyfluoroalkyl substances (PFAS) and cancer: detection methodologies, epidemiological insights, potential carcinogenic mechanisms, and future perspectives. Sci. Total Environ. 953, 176158. 10.1016/j.scitotenv.2024.176158 39255941

