# Supplementary figures and images for: Chain length-dependent mitochondrial toxicity of perfluoroalkyl carboxylic acids: insights from Mito Tox Index evaluation
Source: Front Toxicol. 2025 Jul 15;7:1582891. doi: 10.3389/ftox.2025.1582891 (PMC12303941; doi:10.3389/ftox.2025.1582891)

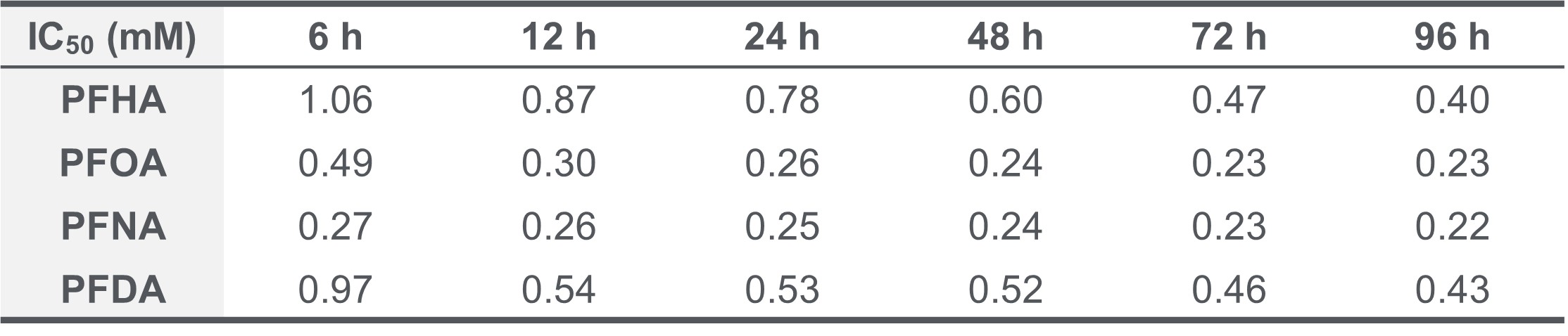

Supplement: Supplementary file 1 [file Image3.jpeg]

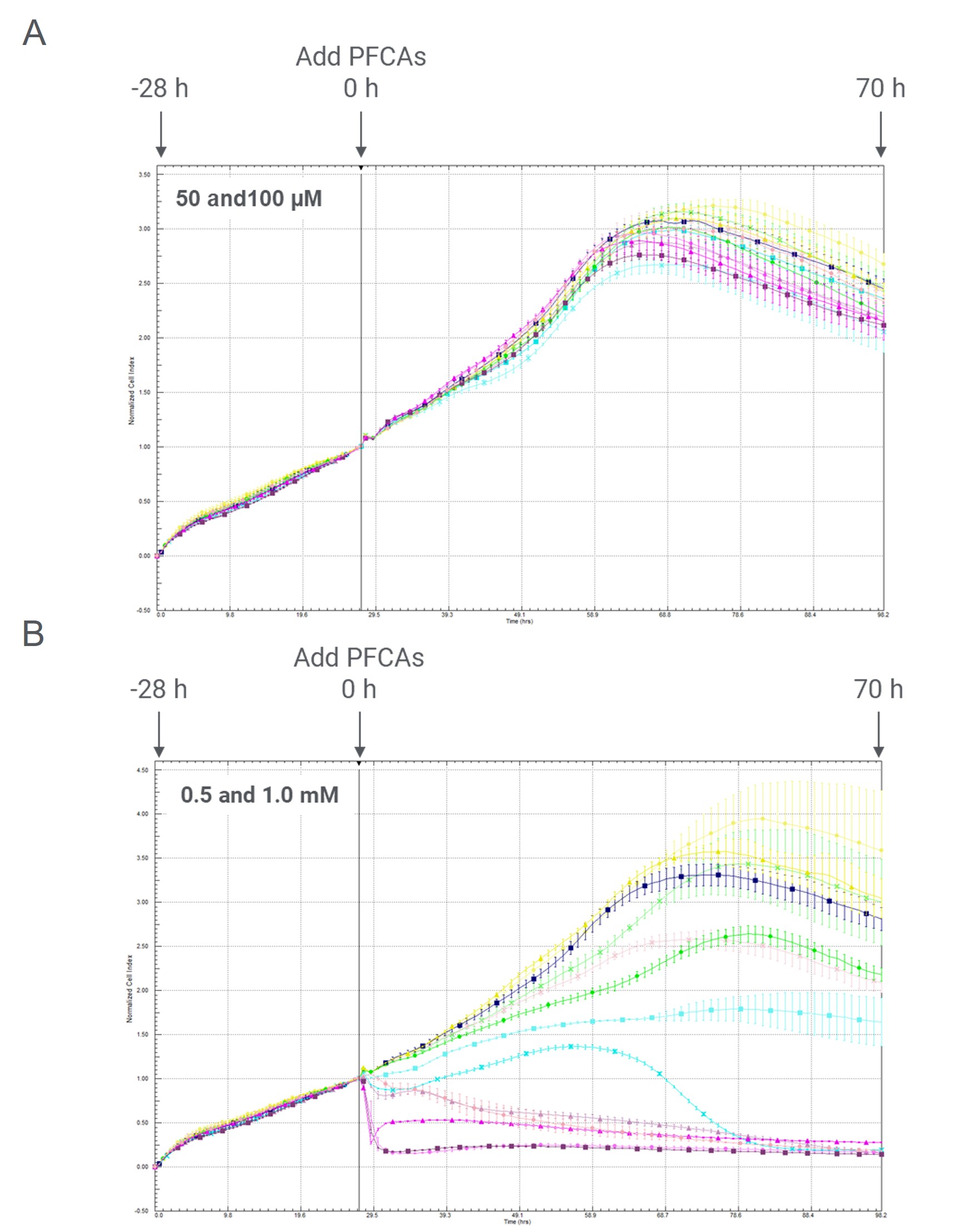

Supplement: Supplementary file 2 [file Image1.jpeg]

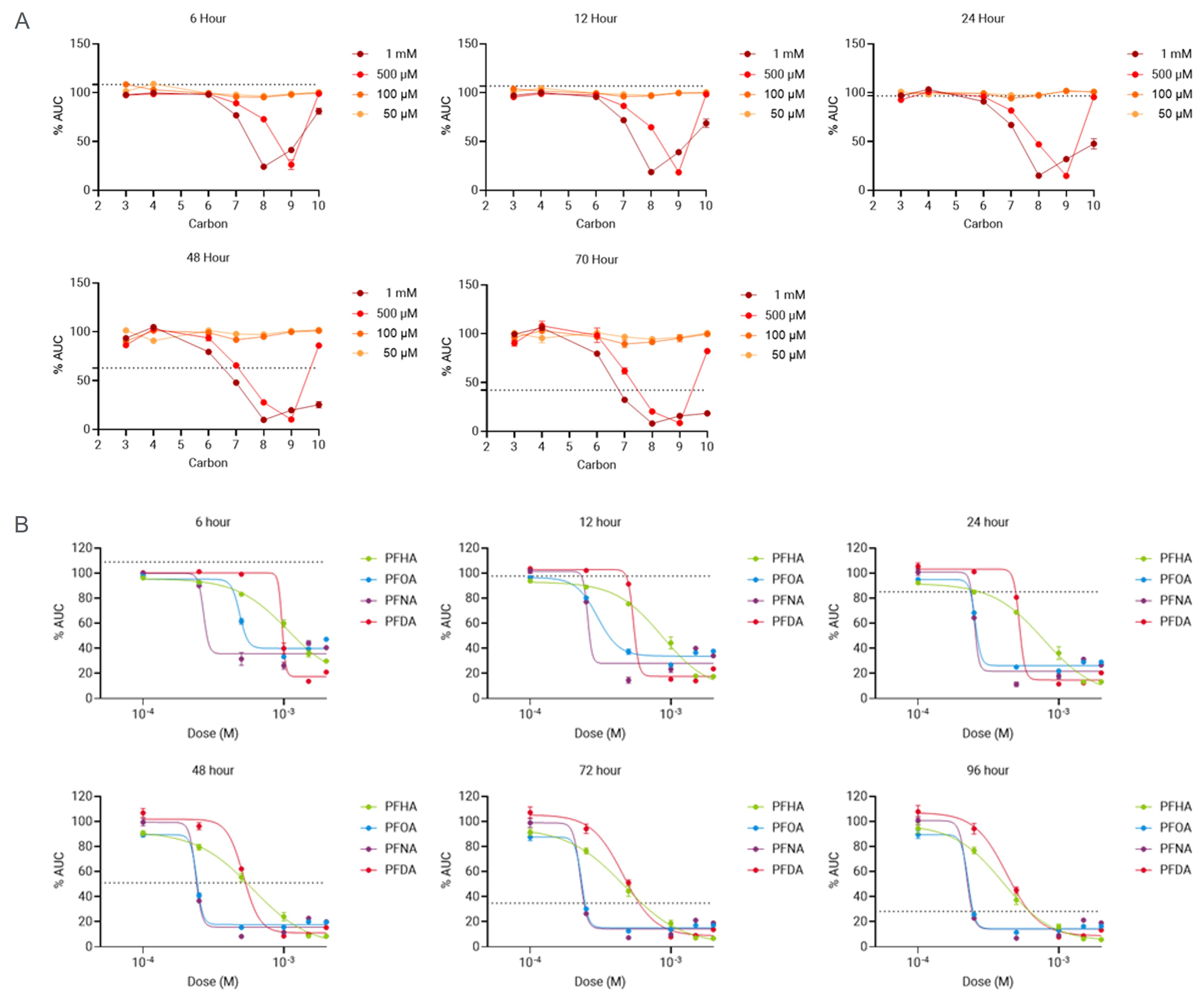

Supplement: Supplementary file 3 [file Image2.jpeg]
